# Defining trait-based microbial strategies with consequences for soil carbon cycling under climate change

**DOI:** 10.1038/s41396-019-0510-0

**Published:** 2019-09-25

**Authors:** Ashish A. Malik, Jennifer B. H. Martiny, Eoin L. Brodie, Adam C. Martiny, Kathleen K. Treseder, Steven D. Allison

**Affiliations:** 1 Department of Ecology & Evolutionary Biology, University of California, Irvine, CA USA; 2 Earth and Environmental Sciences, Lawrence Berkeley National Laboratory, Berkeley, CA USA; 3 Department of Environmental Science, Policy and Management, University of California, Berkeley, CA USA; 4 Department of Earth System Science, University of California, Irvine, CA USA

**Keywords:** Microbial ecology, Soil microbiology, Biogeochemistry, Climate-change impacts, Metabolism

## Introduction

Microorganisms are critical in terrestrial carbon cycling because their growth, activity and interactions with the environment largely control the fate of recent plant carbon inputs as well as protected soil organic carbon [1, 2]. Soil carbon stocks reflect a balance between microbial decomposition of organic carbon and stabilisation of microbial assimilated carbon. The balance can shift under altered environmental conditions [3], and new research suggests that knowledge of microbial physiology may be critical for projecting changes in soil carbon and improving the prognosis of climate change feedbacks [4–7]. Still, predicting the ecosystem implications of microbial processes remains a challenge. Here we argue that this challenge can be met by identifying microbial life history strategies based on an organism’s phenotypic characteristics, or traits, and representing these strategies in ecosystem models.

**Fig. 1:**
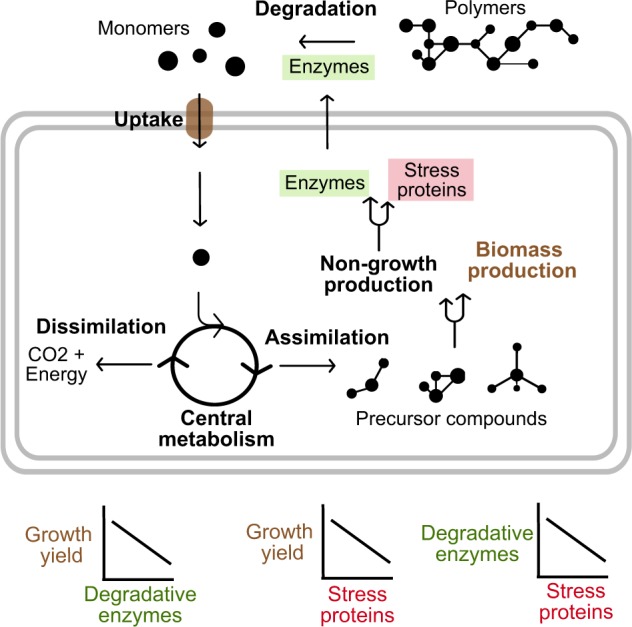
Schematic showing cellular C flux that includes depolymerisation, substrate uptake, assimilation, dissimilation, biomass synthesis and non-growth production. Extracellular enzyme production represents investment in resource acquisition, stress protein production is linked to stress tolerance mechanisms, and biomass production reflects higher growth yield. Forked arrows signify metabolic points where hypothesised tradeoffs in traits might occur. The expected empirical relationships among the key traits are also shown

What are the key microbial traits for soil carbon cycling under environmental change? Microbial growth and survival in soil are impacted by multiple traits that determine responses to varying resource availability and fluctuating abiotic conditions [8]. Cellular maintenance activities (those that do not produce growth) include production of extracellular enzymes to degrade and acquire resources, biomolecular repair mechanisms, maintenance of cellular integrity, osmotic balance, defence, antagonism, cell signalling and motility [9–11]. It is conceivable that microbial investment into maintenance activities would be generally high in soils, with their highly heterogeneous and temporally variable resource distribution and stressful abiotic conditions like extremes of moisture, temperature, pH and salinity [12, 13]. Selective pressures in suboptimal environmental conditions could lead to greater cellular-level physiological allocation to maintenance relative to growth traits (Fig. 1) thereby impacting soil carbon cycling processes.

Life history strategies represent sets of traits that tend to correlate due to physiological or evolutionary tradeoffs, with different strategies favoured under different environmental conditions. For example, metabolic investments in degradative enzyme production for resource acquisition can reduce the efficiency of cellular growth [14, 15]. Furthermore, stress tolerance traits can tradeoff against investment in resource acquisition and growth yield [12, 16–18]. Although some stress tolerance mechanisms may have collateral benefits, the costs must generally be paid at the expense of other physiological processes if resources are limited. Ultimately, microbial metabolic investments and the resulting tradeoffs among traits linked to growth yield, resource acquisition and stress tolerance determine the contribution of microbial processes to ecosystem level carbon fluxes. Thus, information on these microbial traits should be useful in linking microbial processes with ecosystem carbon fluxes [19].

## Life history concepts in ecology

In plant communities, tradeoffs in key fitness traits have been represented through conceptual theories of r- and K-selection, the “leaf economics spectrum” and Grime’s competitor-stress tolerator-ruderal (C-S-R) framework. The r- and K-selection concept recognises two functional groups of organisms: r-selected strategies have short life expectancy and large reproductive effort, whereas K-selected strategies have long life expectancy and invest a smaller proportion of energy and resources into reproduction [20]. Furthermore, leaf economics refers to the resource-driven tradeoffs among leaf traits that regulate plant growth and adaptation to environmental conditions [21, 22]. Grime’s C-S-R triangle is an alternative framework that enumerates three major plant life history strategies: competitors (C) excel at maximising resource capture in productive and undisturbed systems, stress tolerators (S) prevail in continuously low-resource and stressful conditions, and ruderals (R) occupy recently disturbed but less stressful habitats [23]. Such tradeoffs have been shown to apply globally across biomes thus providing the quantitative basis to functionally represent the enormous taxonomic diversity of plant communities in vegetation models [21, 22].

## Applying trait-based theories of life history in microbial ecology

Following on existing ecological theory, microbial ecologists have proposed trait-based classifications of microorganisms. The copiotroph-oligotroph continuum was proposed as analogous to the r- and K-selection theory for plants and animals [24, 25]. Such a classification was mostly based on microbial substrate preferences, trophic strategy and growth rates and has since been widely applied in various environmental contexts [26–29]. Several recent efforts have also applied C-S-R life history strategies to microbial systems, particularly in the context of anthropogenic environmental change [13, 30–32]. Ho et al. [32] classified methane-oxidising bacteria into C-S-R life strategies based on activity, recovery from disturbances, substrate utilisation patterns and stress tolerance. Krause et al. [30] later generalised the same framework for all bacteria while emphasising that additional experiments would be needed to verify the microbial strategies and their underlying traits. Wood et al. [31] justified a microbial C-S-R classification based on different traits derived from predicted functional datasets aimed at assessing the impact of cadmium and influence of the rhizosphere on microbial community assembly. These efforts at applying trait-based concepts in microbial ecology justify additional theory development and experimental evidence to validate the C-S-R framework in microbial ecology.

Although a general theory of life history is attractive, the C-S-R strategies do not necessarily map clearly on to microbial systems. In the plant C-S-R framework, Grime [23] defined habitats based on gradients in disturbance intensity and stress, including multiple abiotic and resource-based factors. These gradients were thought to select for C, S, or R strategies defined by plant traits including morphology, growth form, relative growth rate, leaf longevity, phenology, and seed production. Although some microbial traits like growth rate, biomass turnover, and dormancy may be analogous to plant traits, it can be challenging to apply a plant-based theory to heterotrophic microbes. A major distinguishing factor remains the reliance of heterotrophic microorganisms on an external source of carbon and energy that drive differential cellular allocation and lead to tradeoffs in traits. This makes the quantity and quality of resources in the surrounding environment a key factor in influencing species distribution. In addition, the way microbes encounter and respond to disturbance and stress may not be entirely distinguishable. Thus, it remains unclear how plant-based C-S-R strategies emerge from underlying microbial traits.

Although plant C-S-R strategies do not map well on microbes, it is notable that Grime [23] himself suggested some traits useful in mapping mycelial fungi to the C-S-R strategies: rapid growth and soluble carbohydrate use for ruderals, dense mycelium and rhizomorph production for competitors, and slow growth, persistent mycelium, and low spore production for stress tolerators, which suggests that C-S-R framework may work for mycelial fungi. Moreover, given the vast metabolic diversity of microorganisms and their ability to inhabit extreme environments that are both stressful and frequently disturbed, it is also unclear what dimensionality is needed to adequately describe microbial life history strategies [33]. A framework like the C-S-R life history triangle is a good start in advancing trait-based microbial ecology, while keeping in mind that increasing trait dimensionality may help better predict species distributions.

**Fig. 2:**
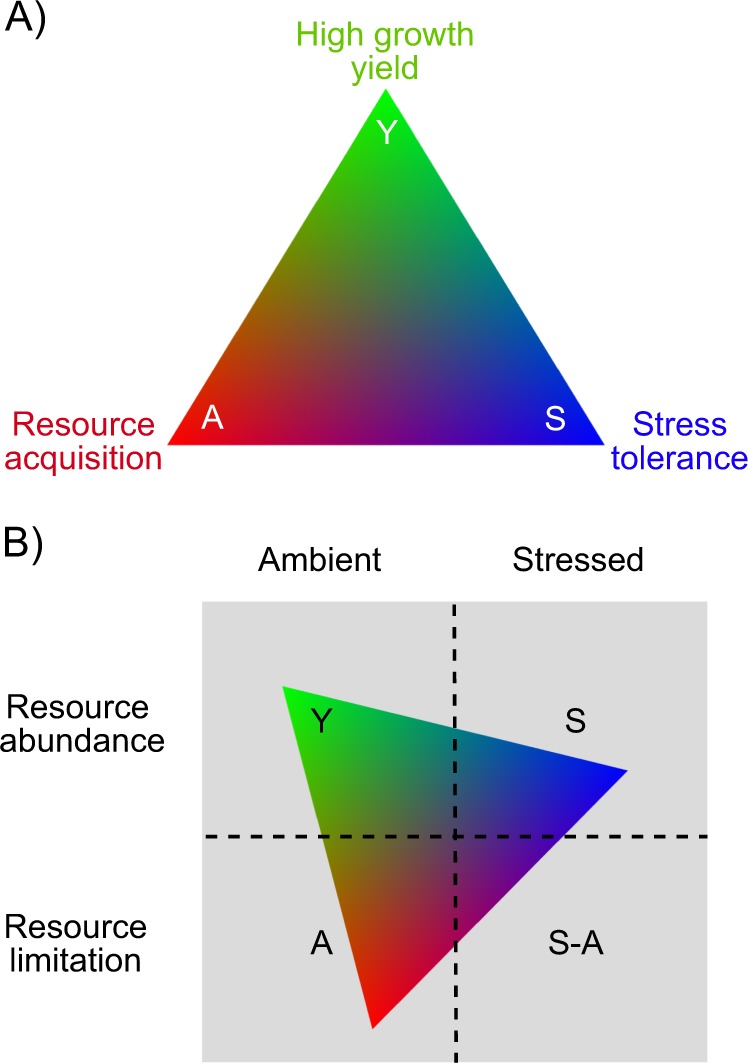
**a** Conceptual figure of microbial Y-A-S life history strategies. High yield (Y): maximises growth efficiency as a result of reduced investments in stress tolerance and resource acquisition; resource acquisition (A): preferential investment in cellular resource acquisition machinery; stress tolerance (S): preferential investment in stress tolerance mechanisms. **b** Hypothesised strategies favoured under particular treatment combinations. The microbial three-dimensional Y-A-S triangle is arrayed on the combinations

Here we propose a revised life history theory for microbes that builds on the work by Wood et al. [31]. Their framework justifies microbial C-S-R classifications based on predicted genomic traits. Traits of the competitor strategy focus on antibiotic production and resource acquisition through siderophores and membrane transporters. Stress tolerator traits relate to damage repair and maintenance of cell integrity. Ruderal microbial traits include investment in processes that promote rapid growth. Wood et al. also define a fourth group of traits related to foraging, such as chemotaxis and flagellum production. Our revised framework emphasises three strategies somewhat analogous to Wood et al.’s version of C-S-R but reclassified into three main microbial life history strategies: high yield (Y), resource acquisition (A) and stress tolerance (S), or Y-A-S along two main axes of environmental variation: resources and abiotic stress (Fig. 2a).

## The high yield (Y) strategy

We define yield, often measured as carbon use efficiency, as the amount of microbial biomass produced per unit of resource consumed [34, 35]. High yield strategists maximise the fraction of resource uptake that is allocated to biosynthetic processes by investing in central metabolism and associated assimilatory pathways such as amino acid, nucleotide, and fatty acid synthesis to build cellular components using these precursor compounds. The absence of resource limitation and stress are expected to favour the high yield strategy (Fig. 2b) [15]. Although parallel to the plant ruderal strategy, the Y strategy is not defined by growth rate, i.e., the change in microbial biomass per unit time [9–11]. In-situ growth rate is not a coherent strategy but rather a complex emergent property that depends on both growth yield and the rate of resource acquisition. In fact, evidence suggests that the growth rate and yield may have a negative relationship [36] or a positive one [37] depending on the system.

The growth rate and yield may also diverge within the Y strategy such that high yields are achieved with different growth rates [15]. Although in-situ growth rate is an emergent property, the maximum potential growth rate (*µ*_max_) is a genome-encoded trait that may be distinct for individual taxa [38]. There is some evidence for a rapid growth, low yield strategy characterised by enhanced metabolism, large cell sizes, high ribosomal production, and high maximum growth rates [36]. However, the strength of the growth rate-yield tradeoff is somewhat inconsistent across individual, population, and community levels [36, 39–41]. Overall, we argue that high yield is a coherent, trait-based strategy whereas high growth rate results from combining any strategy with the right environmental conditions.

## The resource acquisition (A) strategy

Our resource acquisition strategy replaces the plant competitor strategy because microbial competition is mainly over resources. In fact, one could also argue this is true for plants [42]. In soils, microorganisms produce extracellular enzymes to break down complex resources [6, 14, 15] (Table 1). Thus, resource acquisition by heterotrophic microbes depends on uptake of depolymerised substrates using various membrane transporters (Table 1). The level of investment in extracellular enzyme production often reflects substrate status (quality and quantity) of the local environment [43]. Wood et al.’s foraging traits can readily be assimilated into our resource acquisition strategy. However, contrary to Wood et al.’s hypothesis that investment in resource acquisition traits is higher in resource abundant environments like the rhizosphere, we propose that this strategy will prevail in low-resource conditions where microbes would be under selection to increase resource capture at the expense of growth yield. It is also likely that organisms have acquisition strategies that are either uptake optimised (when precursors compounds are readily available in the environment, like in rhizosphere soil or less intensively managed grassland soils) or depolymerisation optimised (when resources are scarce and complex, like in cropland soils) or a combination of both [41, 44, 45].

**Table 1:** Y-A-S strategies, underlying traits and tools to extract trait information

Strategies	Traits	Estimation technique and marker
Growth yield (Y)	Growth per unit resource	- Omics (genomics, transcriptomics, proteomics): markers not known but likely central carbon metabolism, amino acid, fatty acid, and nucleotide synthesis. - Stable isotope tracing into biomass and respired CO_2_* - Biomass and respiration measurements*
Resource acquisition (A)	Degradation of complex substrates	- Extracellular enzyme assays* - Omics: Glycoside hydrolase genes, other CAZy database genes, genes for extracellular enzymes
	Motility: resource discovery	- Omics: genes for flagellar motility, chemotaxis
	Uptake of simple substrates	- Omics: transporters, siderophores
Stress tolerance (S)	Biomolecular damage repair	- Omics: σ factors, molecular chaperons eg. Chaperonin GroEL, DnaK
	Osmolyte production	- Omics + metabolomics*: markers for synthesis of trehalose, glycine betaine, amino acids related to osmotic stress
	Protection from desiccation	- Omics: markers for synthesis of extracellular polysaccharide
	Maintenance of cellular integrity	- Omics: markers for synthesis of cell walls

Asterisk represents quantifiable physiological assays that directly measure phenotypic traits

## The stress tolerator (S) strategy

We adopt Grime’s stress tolerance strategy with little modification because it aligns well across plants and microbes. Soil microbes experience a variety of stressors that change their physio-chemical environment and to which they respond through physiological and evolutionary mechanisms [12, 19]. Specific traits for stress tolerance depend on the kind of abiotic stress experienced by microbial communities. Regardless of the form of stress imposed, certain global patterns in phenotypic expression are common, including σ factors or molecular chaperons aimed to minimise or mitigate biomolecular damage [31, 46–48] (Table 1). In cases such as high acidity or salinity, microbes employ various strategies to maintain cellular integrity and osmotic balance through changes in the structure and composition of cell envelopes [49]. Under drought scenarios, stress tolerance strategies involve production of osmolytes like trehalose and glycine betaine or synthesis of extracellular polymeric substances (EPS)—usually polysaccharides—to protect cells from desiccation (Table 1) [12, 50]. Thus, microbes exposed to suboptimal abiotic conditions would possess traits linked to stress tolerance at the expense of other traits.

## Strategies under varying conditions

Tradeoffs in resource allocation should prevent microbes from excelling at multiple Y-A-S strategies. Different strategies should be favoured under different environmental conditions arising from spatial or temporal variability in resource status and abiotic conditions (Fig. 2b). For example, drought should select for S-strategists that increase investment in osmolyte production to maintain cellular osmolarity. Osmolyte production is energetically very expensive and reduces growth yield [12]. In environments with high availability of polymeric resources (e.g. polysaccharides) but fewer simple resources (e.g. simple sugars, amino acids), depolymerisation-optimised A-strategists should outcompete Y-strategists by investing in extracellular enzyme machinery. Thus, abiotic stress or resource limitation should select against Y-strategies because of a need for investment in costly resource acquisition or stress tolerance mechanisms. Thus, A-strategists catalyse polymer decomposition and soil carbon loss, whereas Y-strategists may convert monomeric substrates into microbial residues that can then contribute to organic matter stabilisation [7, 41, 51]. These examples illustrate how life history tradeoffs can have consequences for soil carbon dynamics.

## Approaches for measuring and testing Y-A-S strategies

Technological innovations like next generation sequencing have massively improved our understanding of the taxonomic and functional diversity of soil microbial communities and their shifts in response to anthropogenic influences [13]. Current approaches have mostly focused on identifying taxonomic and functional responses to environmental changes. However, integration of these large microbial molecular datasets with process rate measurements remains a challenge, thereby making it difficult to link microbial composition and function with ecosystem processes [19, 30, 52]. More efforts are needed to determine how microbial genomic information translates into traits that influence abundance, metabolite production, and ultimately carbon cycling rates in ecosystems.

Omics datasets on genes, transcripts, proteins and metabolites can be used to quantify the traits that define our Y-A-S strategies [13, 30]. Population-level trait assessment will help validate genotype-phenotype linkages and enable quantification of Y-A-S life history strategies at the community level. Trait information from populations can be gathered from sequenced genomes where cultured microbial strains are available [44]. An increasing number of studies are reporting new media and culturing conditions to isolate previously uncultured microbes which should increase the diversity of populations in databases. Data can also be extracted from existing databases that link taxonomy, phylogeny, or specific genes to measured traits and environmental preferences [53, 54]. In other cases, individual population genomes can be assembled from culture-independent shotgun metagenomic datasets; this novel approach is gaining popularity as it facilitates physiological investigations of hitherto uncultured taxa [55].

At the community level, traits integrate tradeoffs across phylogenetically undifferentiated populations [10]. Spatial competition, priority effects and other community level processes will also structure populations and affect processes. Community-aggregated measurements have the potential to predict microbial processes that drive ecosystem fluxes [56]. However, such aggregated traits obtained through simple summation of individual taxon traits may not reflect real process rates [19, 52]. Quantifying phenotypic traits directly using physiological assays can help overcome this issue.

## Omics and physiological techniques to quantify traits

Growth yield (synonymous with growth efficiency) is a challenging property to extract from omics datasets because we still do not understand its genetic determinants. However, there are quantitative methods for estimating growth yield and its components (Table 1, Geyer et al. [10]). Approaches include measuring the change in biomass proxies and respiratory loss or following a tracer—commonly a stable isotope—in cellular fractions. Yield is often measured as the proportion of C substrate invested into biomass relative to that lost through respiration. Recent studies emphasise, though, that growth yield is actually an emergent and dynamic property of multiple underlying traits related to cellular maintenance, protein synthesis and export, cellular stoichiometry, electron transport chain and respiratory pathways [11, 34, 57].

This complexity creates challenges for extracting trait information on growth yield from omics datasets. Still, several genes/transcripts/proteins linked to central carbon metabolism and associated assimilatory pathways have been found to correlate with high yield (Y) strategy. For example, markers of pathways such as ribosomal protein synthesis, amino acid synthesis, nucleotide and fatty acid metabolism could be linked to growth but may not necessarily be an indication of an efficient physiology [36, 41]. Similarly, increased abundance of biogenic amino acids and nucleotides in intracellular metabolomic profiles when corrected for biomass may provide an indication of cellular growth yield [44, 58, 59]. Increased respiration associated with enzyme production (A strategy) or maintenance of cellular integrity (S strategy) should directly and negatively affect measured growth yield. Metabolic pathways based on alternative electron acceptors are often a characteristic of extreme environments and can lead to low growth yields [11].

Resource acquisition traits have been estimated with omics and biochemical techniques at both the population and community levels (Table 1). Extracellular enzyme assays provide estimates of microbial enzyme activity and the potential to degrade various complex substrates. Genes and transcripts encoding these enzymes can also be predicted from omics datasets. In addition, there is growing interest in linking Carbohydrate-Active Enzyme (CAZy) database genes to microbial substrate degradation and resource acquisition potential. The CAZy database includes genes that code for enzymes that synthesise and break down complex carbohydrates and glycoconjugates [60]. For example, glycoside hydrolases (GH) are involved in plant cell wall degradation and act on glycosidic bonds between carbohydrates or between carbohydrates and non-carbohydrate moieties [61, 62]. In soils with lower resource availability, GH genes are expected to be more diverse and abundant, potentially leading to higher GH enzyme activities [44, 45]. Once the complex polymers are degraded into simpler molecules, they are taken up by transporters (Fig. 1). A variety of transporters, particularly the ATP-binding cassette transporters (ABC-transporters), with differential substrate specificity can also be predicted from omics datasets. Greater investment in uptake transporters has been observed in root-associated microorganisms with plentiful substrates allowing the cells to reduce investment into extracellular enzyme production and increase their growth yield [44, 48].

Stress tolerance traits in the form of σ factors, molecular chaperons or specific physiological adaptations can be extracted from widely used omics tools. For example, increased frequency and diversity of chaperons has been observed in soil communities from intensive land use treatments, demonstrating the cellular need for biomolecular repair in such degraded soils [41, 46]. Some low molecular weight metabolites synthesised in response to environmental stimuli (e.g. trehalose as an osmolyte under drought stress) can be quantified using mass spectrometry tools like LC-MS and FT-ICRMS [50, 59, 63]. Promising genetic indicators for communities under drought stress include genes for synthesis of osmolytes like trehalose, glycine betaine, choline, and ectoine as well as genes for extracellular polymeric substance (EPS) and capsular synthesis that help form “sponges” to retain water in cell envelopes.

## Carbon cycling implications of tradeoffs in Y-A-S traits

We posit that microbial metabolic investments and the resulting tradeoffs among key traits determine the contribution of microbial processes to ecosystem-level carbon fluxes. These traits will interact with abiotic factors such as microbial residue chemistry, mineral composition, and aggregate structure to determine long-term organic matter storage in soils. Microbial physiological responses and the resulting effects on growth yield can affect carbon balance through two main mechanisms. On the one hand, microbial biomass is thought to contribute significantly to organic matter accumulation and hence to the genesis of soil organic matter [1, 41, 51]. On the other hand, microbial biomass and extracellular enzymes contribute to plant litter and soil organic matter degradation. Under our Y-A-S framework, Y-strategists with increased investment into growth and biomass production would contribute to microbial residue formation that can be stabilised through organo–mineral interactions or aggregation. In contrast, A-strategies should contribute more to decomposition and carbon loss through investment in extracellular enzyme production [2, 51]. Selection for A-strategists could also occur under a lower organic matter environment that stimulates enzyme production to mine resources [45]. Carbon impacts of S-strategists might depend on the type of stress compounds produced, with more complex compounds like EPS contributing more to carbon storage than simple compounds like osmolytes [12, 50]. By diverting investments away from growth, S-strategists could also reduce soil carbon accumulation. The effect of microbial physiological adaptation to climate change and its consequences for soil C cycling could thus be determined by assessing shifts in microbial Y-A-S life history strategies.

## Approaches to modelling Y-A-S strategies to predict carbon fluxes

Representing microbial diversity has been a big challenge for models projecting ecosystem responses to environmental change [19]. This challenge introduces uncertainty that affects model predictions of future climatic change [4, 64]. Such uncertainties imply a need for better mechanistic models, and improved representation of microbial diversity and physiology could increase the accuracy of projected soil carbon fluxes. Although helpful, taxonomic information has limited utility in predicting ecosystem processes without information about the functional traits of the taxa present in a community. This decoupling arises due to various confounding factors such as functional redundancy, dormancy, phenotypic plasticity, etc. associated with the complexity of microbial characteristics across space and time [13, 19, 52]. Thus, focussing on life history strategies arising from the interactions between microbial populations and the environment should help better link microbial ecology with ecosystem pools and fluxes.

Previously, functional groups have been incorporated into ecosystem models like the MIcrobial-MIneral Carbon Stabilisation (MIMICS) model to predict the biogeochemical response of soil organic matter decomposition and stabilisation [5]. In this model, copiotrophic and oligotrophic functional groups represent fast-growing low yield and slow-growing high yield strategists, respectively. However, traits for acquisition of complex resources and tolerance to abiotic stressors are difficult to incorporate into the copiotrophic-oligotrophic dichotomy [2, 12]. To better capture the metabolic flexibility of soil microbial populations, MIMICS could add stress tolerance and resource acquisition traits and represent the tradeoffs between these maintenance traits and the different existing growth strategies [13, 30].

Adding a third dimension may help achieve the level of complexity required to represent the metabolic diversity of microbial populations. A trait-based modelling framework based on microbial Y-A-S strategies holds promise for representing microbial characteristics in simulations of system-level processes at various spatial scales [19]. The cellular mechanisms underlying tradeoffs in key traits can be incorporated into microbial functional models like MIMICS to reveal how these tradeoffs structure microbial communities and their resulting carbon cycle functions. This advance could be achieved by incorporating Y-, A- and S-functional groups into models. Alternatively, explicit microbial representation may not be required to accurately model ecosystem functions [4, 19, 65]. At larger scales, microbial community composition and physiology may be a response to changes in resources and abiotic conditions rather than an independent driver of processes. To this end, we suggest that growth yield can be a master response trait in ecosystem models that integrates the physiological processes of populations comprising communities [4, 57].

**Fig. 3:**
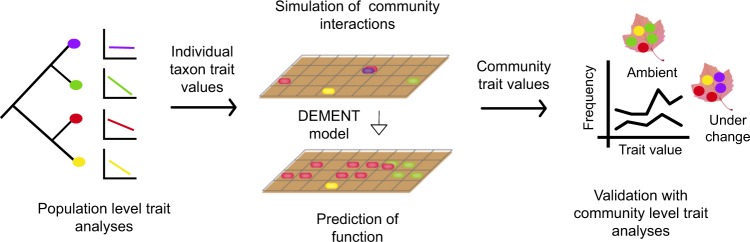
Summary of the proposed trait-based framework incorporating microbial life history strategies into the DEMENT model to predict community response and its ecosystem consequences under environmental change (adapted from Allison and Goulden [69])

Models representing continuous variation in traits across taxa are also promising tools for predicting biogeochemical processes based on the Y-A-S framework. For example, DEMENT is a local scale, trait-based model that simulates litter decomposition and soil carbon transformations by diverse microbial communities [66, 67]. The model uses relationships between Y and A traits as a mechanistic basis for predicting how microbial communities and carbon cycling processes will respond to future environmental change (Fig. 3, [68]). Yield in the model is a function of multiple factors, including substrate type and stoichiometry, enzyme production rates, uptake investment, and temperature. The most recent version of DEMENT also includes a simple representation of drought stress tolerance [69]. After incorporating trait tradeoffs derived from omics or other data sources for individual taxa, DEMENT projects community responses and carbon cycling consequences under simulated environmental conditions. Model outputs can be validated with in-situ trait distributions at a community level or with ecosystem processes like organic matter decomposition rates (Fig. 3) [66, 69]. This validation approach can also be applied to other individual-based models that simulate spatial structuring of microbial populations based on functional groups characterised by traits [70, 71]. Based on the successful trait-based modelling of global vegetation, one could expect rapid progress in developing models that incorporate microbial traits.

## Conclusions

There is growing interest in applying trait-based concepts to predict the microbial mechanisms driving global biogeochemical cycles. By adapting several theories from plant ecology, we define microbial high yield, resource acquisition, and stress tolerator strategies based on key traits that are linked to organismal fitness. Our Y-A-S framework is one testable alternative for organising life history strategies of microbes. We recognise that other useful frameworks may also be proposed. Still, there is good evidence that growth yield, resource acquisition, and stress tolerance strategies encompass many of the key traits that regulate microbial community functioning and appear in microbial-explicit models. Therefore, we envisage our Y-A-S framework will guide new empirical and modelling studies on the mechanisms driving soil carbon fluxes. We anticipate that these approaches will improve our understanding of the physiological constraints facing microbes under anthropogenic influence. By linking population-level response traits to community and ecosystem processes, our life history theory can improve predictive understanding of soil C responses to future climatic change.
